# Unplanned Postoperative Angiography After Isolated Coronary Artery Bypass Grafting: State of the Art and Future Perspective

**DOI:** 10.3390/medicina61071241

**Published:** 2025-07-09

**Authors:** Konrad Wisniewski, Giovanni Concistrè, Angelo Maria Dell’Aquila

**Affiliations:** 1 Department of Cardiothoracic Surgery, University Hospital Muenster, 48149 Muenster, Germany; konrad.wisniewski@ukmuenster.de; 2 Department of Adult Cardiac Surgery, Gaetano Pasquinucci Heart Hospital, Fondazione CNR-G. Monasterio, 54100 Massa, Italy; concistr@ftgm.it; 3 Department of Cardiac Surgery, University Hospital Halle, 06120 Halle, Germany

**Keywords:** postoperative myocardial ischemia, coronary artery bypass grafting, unplanned postoperative angiography

## Abstract

Unplanned postoperative coronary angiography (uCAG) following isolated coronary artery bypass grafting (CABG) represents a significant clinical challenge, reflecting postoperative myocardial ischemia (PMI) with substantial impact on outcomes. The incidence of uCAG varies from 0.39 to 5.3%, depending on institutional protocols and diagnostic thresholds. Elevated cardiac biomarkers (high-sensitivity troponin and CK-MB), ECG changes, and hemodynamic instability are key indicators guiding uCAG. While associated with increased short-term mortality and morbidity, timely identification and treatment of graft-related complications via uCAG can improve midterm survival. Percutaneous coronary intervention (PCI) often emerges as the preferred therapeutic strategy over redo CABG. Future efforts should focus on refining risk stratification models, expanding the role of non-invasive imaging modalities, and validating early intervention strategies through prospective studies. Establishing standardized criteria for diagnosing and managing PMI remains critical to enhance outcomes and healthcare efficiency.

## 1. Introduction

Coronary bypass surgery is one of the most frequently performed surgeries worldwide [1,2]. Despite a slight decline in developed countries, the number of coronary artery bypass grafting (CABG) procedures has stabilized [3]. CABG remains a cornerstone treatment for severe coronary artery disease. Despite advancements in surgical techniques and perioperative care, a small number of patients experience postoperative myocardial infarction (PMI) in the postoperative period [4].

The Fourth Universal Definition of Myocardial Infarction (2018) provides clear criteria for the diagnosis of PMI after CABG. According to this definition, PMI is diagnosed when there is an elevation of cardiac troponin values >10 times the 99th percentile upper reference limit (URL) within 48 h after surgery, in conjunction with either new pathological Q waves or new left bundle branch block, angiographic documentation of a new graft or native coronary artery occlusion, or imaging evidence of new loss of viable myocardium or new regional wall motion abnormality [4,5].

Clinically, PMI can ultimately undermine results and limit early and late survival [4]. The incidence of PMI ranges from 0.3 to 9.8% after isolated CABG and from 0.7 to 11.8% after concomitant valvular surgery [4]. Consequently, increased mortality rates vary from 5.1 to 24%. In a meta-analysis by Biancari et al., the mortality rate after PMI was 8.9%, substantially higher than in patients without PMI (1.8%) [6]. Numerous intraoperative factors can cause PMI [4]. Treatment options to attenuate signs of ongoing ischemia are still not standardized. Among them, unplanned coronary angiography (uCAG) offers the possibility of identifying the cause of PMI and guiding appropriate treatment. This literature review provides a comprehensive overview of the current knowledge regarding uCAG after PMI, highlighting indications, diagnostic value, therapeutic impact, and outcomes while examining future directions for improving patient care and clinical decision making.

## 2. Literature Search Methods

We performed a narrative literature review to identify studies related to unplanned postoperative coronary angiography after coronary artery bypass grafting. We searched PubMed, Scopus, ScienceDirect, and Google Scholar for articles published between January 2000 and January 2025. The search terms included “coronary artery bypass grafting”, “unplanned coronary angiography”, “re-angiography”, “emergency coronary angiography”, “perioperative myocardial infarction”, and “postoperative myocardial ischemia”, “PMI”. Inclusion criteria comprised English-language original articles, reviews, meta-analyses, and clinical guidelines reporting data on the incidence, diagnosis, management, and outcomes of uCAG after CABG. After screening, more than a dozen articles focusing specifically on uCAG after CABG were selected, with a total of 27 topic-related articles included in the final review.

## 3. Definition and Incidence of Unplanned Coronary Angiography

Unplanned CAG constitutes an invasive diagnostic procedure conducted to evaluate myocardial ischemia during the postoperative phase subsequent to CABG [7]. This procedure entails the injection of contrast dye into the coronary arteries to visualize blood flow and detect any obstructions or abnormalities that may be inducing ischemia. In contrast to elective or planned angiography, uCAG is performed on an urgent basis due to the abrupt onset or exacerbation of symptoms indicative of myocardial ischemia. Unplanned CAG is distinct from planned angiography, which may form part of a hybrid revascularization strategy [7]. Unplanned CAG is conducted explicitly in response to unexpected ischemic events that arise following CABG, necessitating immediate assessment.

The main reason for uCAG is suspected PMI [7]. This condition refers to myocardial damage that happens during or soon after CABG, typically due to graft failure, incomplete revascularization, or other technical issues. If PMI is suspected due to clinical manifestations, ECG alterations, or raised cardiac biomarkers, CAG is necessary to identify the root cause and inform suitable management approaches.

The reported incidence of uCAG following isolated CABG varies significantly between institutions, ranging from 0.39 to 5.3% [2,4,7,8,9,10,11]. This variability likely reflects differences in patient populations, surgical techniques, diagnostic criteria, and institutional practices. A study by Heuts et al. [7] indicated that 4.1% of patients undergoing isolated non-emergency CABG required postoperative CAG within 30 days after surgery, excluding planned hybrid revascularization procedures. In contrast, Norman et al. found a considerably lower incidence of 0.39% in patients undergoing isolated CABG requiring unplanned cardiac catheterization [2]. This discrepancy could be attributed to differences in study populations, timeframes, and specific criteria used to define uCAG, noting that most catheterizations occurred within the first postoperative week. Davierwala et al. reported a higher incidence of 5.3% for uCAG, indicating considerable variability and emphasizing its clinical significance [12]. Preußer et al. reported a 4.2% incidence rate, further underscoring the variability in reported figures and the importance of stringent diagnostic criteria [13]. Additionally, a meta-analysis conducted by Biancari et al. that comprised multiple studies and diverse populations identified a pooled incidence rate of approximately 4.4%, reflecting variability across different clinical settings and methodologies [6]. Table 1 summarizes key published data on uCAG incidence in recent studies.

Differences in incidence rates of uCAG likely reflect varying diagnostic thresholds, particularly the broader use of biochemical criteria. For example, Davierwala et al. used creatine kinase-MB elevations (>4× upper limit of normal) as an independent indication for angiography [12]. Preußer’s study observed distinct cardiac enzyme kinetics: infarction patients peaked at 12–24 h post-CABG, while non-infarction cases peaked immediately postoperatively and declined thereafter [13]. PMI is a notable complication following CABG surgery, with reported incidences varying across studies. One study reported an incidence of PMI of 5.3% in their patient population [12]. Another study reported a similar incidence of PMI of 4.2% [13]. The diagnosis of PMI typically involves assessing postoperative cardiac enzyme elevation, new electrocardiographic signs of myocardial ischemia, and new regional wall motion abnormalities. The mortality rate associated with PMI ranges from 7 to 16% [13]. The lack of standardized definitions and criteria for diagnosing PMI contributes substantially to these variations in reported incidence rates. Institutional differences in thresholds for performing angiography for suspected PMI further influence these discrepancies. Unplanned CAG remains essential for identifying the underlying causes of PMI, critically guiding treatment decisions and influencing patient outcomes.

Investigating unplanned CAG is crucial due to its significant implications for patient outcomes, healthcare costs, and the overall effectiveness of CABG procedures. Several studies have highlighted the adverse consequences associated with unplanned CAG, emphasizing the need for a better understanding of its causes, predictors, and optimal management strategies.

## 4. Indications and Diagnostic Criteria for Unplanned Coronary Angiography

The diagnosis of PMI after CABG relies on a combination of clinical, electrocardiographic, biomarker, and imaging findings. Challenges in diagnosis arise due to perioperative shifts in biomarker levels and the non-specificity of some ECG changes in the postoperative setting. Therefore, an integrated approach is essential, using all available modalities to distinguish true myocardial injury from benign perioperative changes.

### 4.1. Clinical Presentation of PMI and Indication to Unplanned Coronary Angiography

PMI manifests through various clinical signs and symptoms, including ECG changes, regional wall motion abnormalities in echocardiography, biomarker elevations, and hemodynamic instability [4,15]. Such indicators prompt clinicians investigating uCAG to evaluate suspected ischemia causes comprehensively.

Electrocardiogram (ECG) abnormalities, including ST-segment elevation or depression, new-onset Q waves, or significant T-wave inversion, are critical indicators for PMI [4]. Davierwala et al. highlighted ECG changes as a primary criterion for considering immediate postoperative angiography, especially when coupled with clinical deterioration or ongoing ischemic symptoms [12]. Similarly, Preußer et al. emphasized that new or progressive ECG abnormalities strongly correlate with graft occlusion or significant stenosis, necessitating rapid diagnostic evaluation via CAG [13].

Echocardiography can detect new regional wall motion abnormalities suggestive of ischemia [18]. By visualizing the contraction of the heart muscle, echocardiography can identify areas where the wall motion is impaired, indicating reduced blood flow. Although transthoracic echocardiography is a non-invasive and readily available diagnostic tool, it may not always be sensitive enough to detect subtle ischemia or graft failure.

Cardiac biomarkers play an essential role in diagnosing PMI. Elevations in CK-MB and cardiac troponin levels serve as quantifiable markers of myocardial injury. High-sensitivity cardiac troponin I (hs-cTnI) has shown significant diagnostic accuracy with values exceeding 8000 ng/L within 12–16 h postoperatively, strongly correlating with graft failures requiring intervention [15,19]. According to recent literature, a secondary rise of CK-MB levels exceeding 7 mg/L within 72 h post-surgery is associated with high sensitivity (94%) and specificity (80%) for PMI detection [7,20]. This secondary elevation of CK-MB indicates ongoing myocardial injury beyond the initial surgical trauma.

Elevated postoperative cardiac biomarkers, especially high-sensitivity troponins and CK-MB, predict adverse outcomes and the need for CAG [21]. Early troponin elevations (>10× upper reference limit) indicate significant PMI, warranting further investigation [7,19,20]. CK-MB elevations of a 3–10× upper reference limit are traditionally used as diagnostic thresholds, but variability exists across guidelines [7]. Troponins offer higher specificity compared to CK-MB [20,22,23].

Hemodynamic instability, including significant hypotension, malignant arrhythmias, heart failure, or cardiac arrest, also constitutes critical indications for urgent CAG [4]. Such instability usually reflects significant myocardial ischemia or graft compromise, prompting rapid angiographic assessment to identify reversible ischemic causes and guide appropriate therapeutic interventions [12,13]. The absence of universally accepted thresholds for biomarker elevation or ECG criteria continues to challenge PMI diagnosis standardization. This variability leads to differences in clinical practice and decision-making thresholds across institutions. Standardizing these diagnostic criteria remains essential for future research and clinical guideline development.

Coronary angiography remains the gold standard for identifying graft or native coronary occlusion [18]. By directly visualizing the coronary arteries and bypass grafts, angiography can detect stenosis, thrombosis, or other abnormalities that may be causing ischemia. Furthermore, angiography enables immediate intervention to restore blood flow.

### 4.2. Challenges in Defining and Diagnosing Postoperative Myocardial Ischemia

Despite the availability of various diagnostic modalities, challenges remain in defining and diagnosing PMI. As highlighted in recent guidelines, the diagnosis of PMI after CABG should adhere to the criteria of the Fourth Universal Definition of Myocardial Infarction, which requires a postoperative increase in cardiac troponin I (cTnI) or troponin T (cTnT) exceeding 10 times the 99th percentile URL within 48 h, along with corroborating clinical, electrocardiographic, angiographic, or imaging evidence. The adoption of these criteria is crucial for consistency in both clinical practice and research [4,5].

The diagnosis of postoperative myocardial infarction after CABG remains challenging due to variability in definitions across studies and clinical practice [7]. While the Fourth Universal Definition of Myocardial Infarction now provides standardized criteria, clinical application is often complicated by postoperative changes in cardiac biomarkers, the non-specificity of some ECG alterations, and variable imaging findings. Therefore, a comprehensive approach is required to achieve accurate diagnosis and appropriate management.

Distinguishing between reversible and irreversible causes of PMI is crucial for guiding effective interventions. Reversible causes, such as graft spasm or hypovolemia, may respond to medical management, while irreversible causes, such as graft thrombosis, typically require revascularization. Angiography can help differentiate between these causes and guide appropriate treatment decisions.

Overreliance on biomarker elevations alone may lead to unnecessary invasive procedures [22]. While elevated cardiac biomarkers are indicative of myocardial injury, they do not always reflect clinically significant ischemia. In some cases, biomarker elevations may result from non-ischemic causes, such as surgical trauma or inflammation.

Therefore, it is essential to interpret biomarker results within the context of the patient’s clinical presentation and other diagnostic findings [21].

## 5. Etiology and Mechanisms of Postoperative Myocardial Ischemia

PMI following CABG can result from failures related to grafts, patient comorbidities, and perioperative inflammatory responses. Demographic characteristics such as female sex, younger age, and smaller body size have also been linked to higher graft failure rates. Inflammatory and thrombotic mechanisms are believed to play a central role, with the perioperative inflammatory response promoting platelet activation and graft occlusion. Prevention thus requires not only precise surgical technique but also careful management of patient comorbidities and perioperative pharmacologic therapy.

### 5.1. Technical Factors Related to Graft Failure

**Figure 1:**
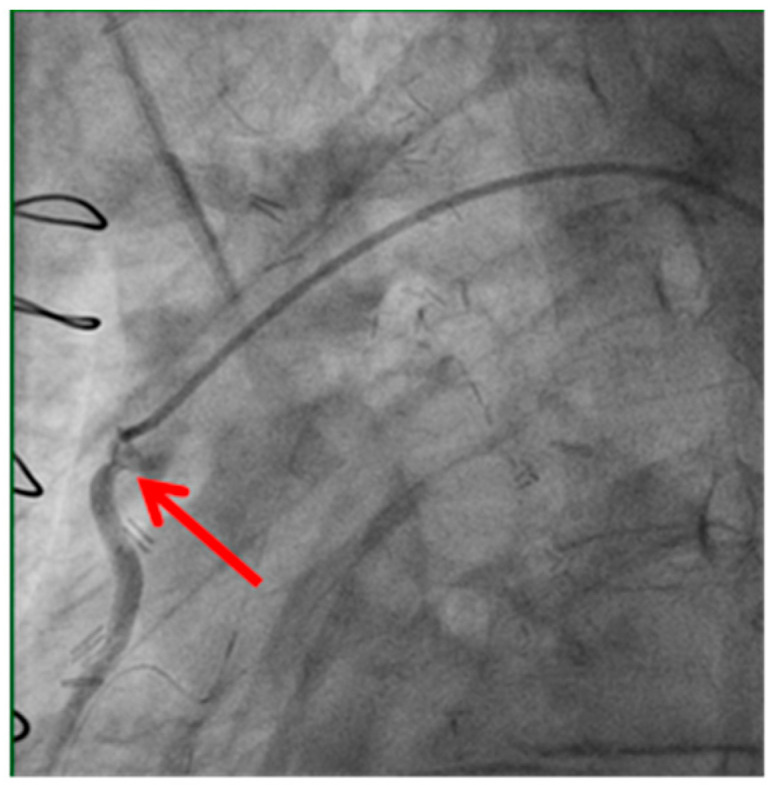
Stenosis of proximal anastomosis.

**Figure 2:**
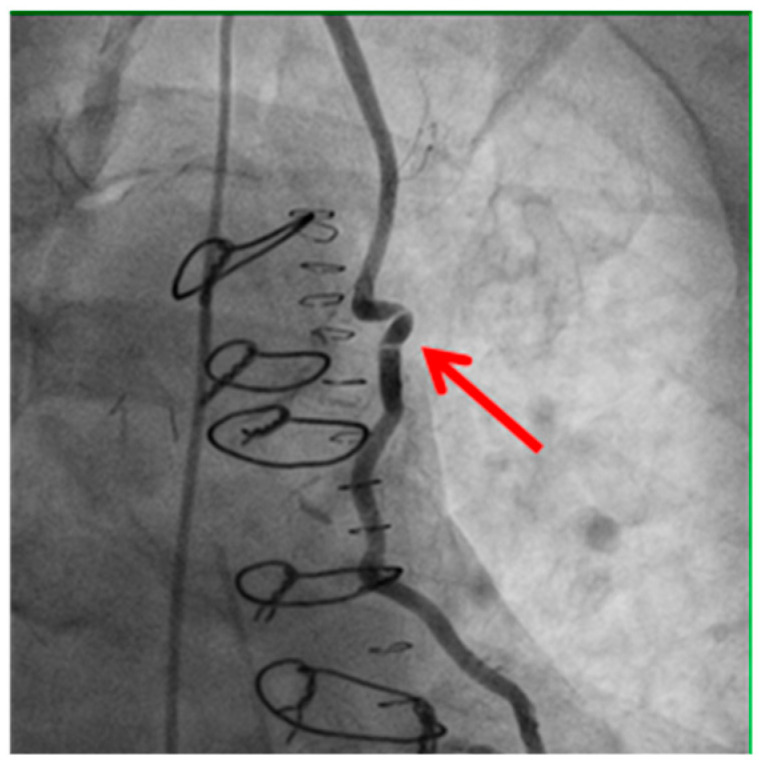
Kinking of the bypass graft.

**Figure 3:**
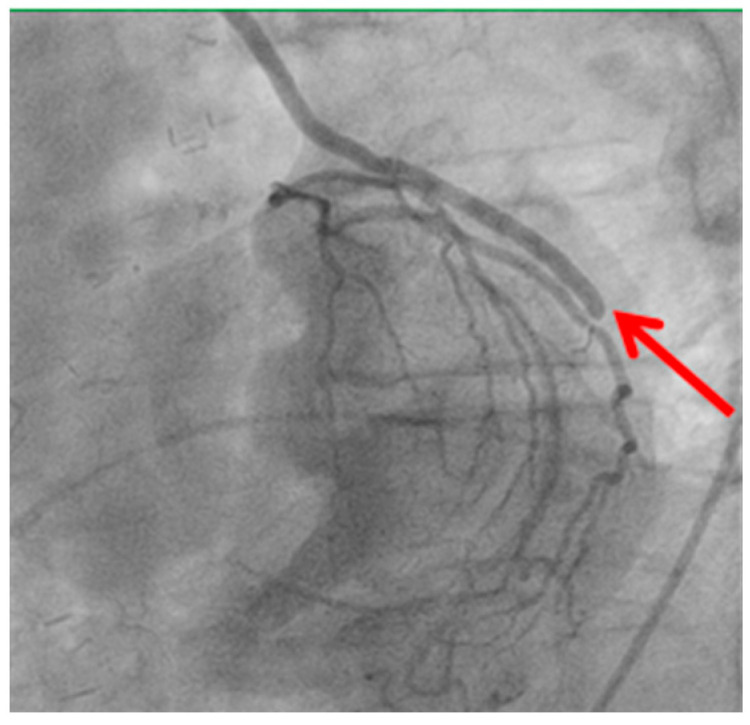
Stenosis of distal anastomosis.

**Figure 4:**
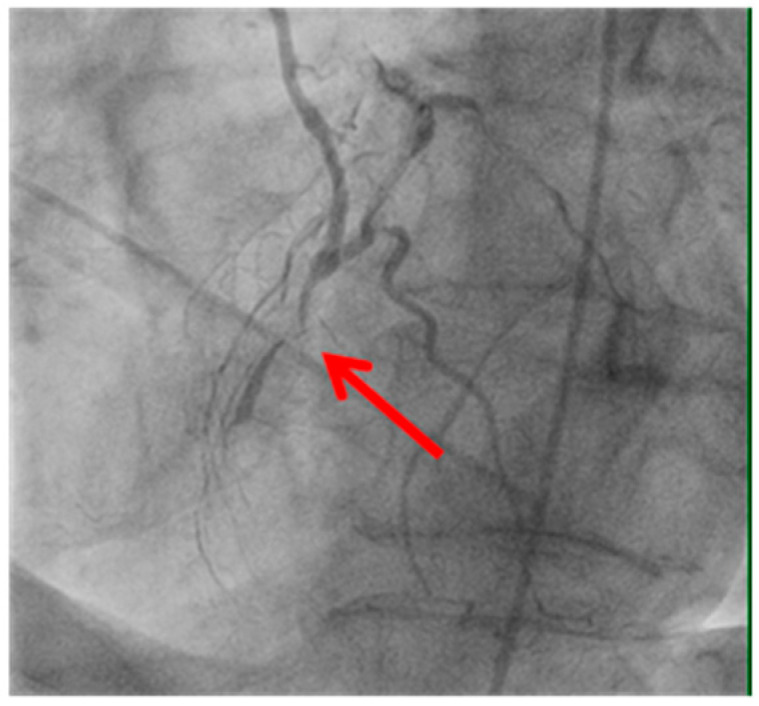
Anastomosis performed before the vessel stenosis.

Technical issues remain a principal cause of PMI, with early graft thrombosis or occlusion being leading indications for uCAG (Figure 1, Figure 2, Figure 3 and Figure 4 illustrate typical technical causes, including proximal anastomotic stenosis, kinking, distal anastomotic stenosis, and misplaced anastomoses). Notable contributors include anastomotic stenosis, kinking, overstretching of grafts, and temporary graft spasm [13,21]. Prueßer et al. demonstrated that 30% of PMI cases detected via CAG were graft-related failures [13]. In this context, uCAG often reveals technical failures immediately post-surgery, providing the advantage of guiding targeted percutaneous coronary intervention or revascularization. Graft spasm, which can be relieved by nitrates, may also lead to acute ischemia. It refers to the temporary constriction of the bypass graft, reducing blood flow to the myocardium. This condition can be caused by various factors, including surgical trauma, inflammation, or the release of vasoactive substances [21].

Stenosis of the proximal anastomosis (shown by the red arrow) visualized on early postoperative coronary angiography. This image demonstrates a technical cause of early graft failure, which can lead to perioperative myocardial infarction if not promptly identified and treated.

Example of bypass graft kinking (shown by the red arrow) detected on angiography. Kinking may compromise graft patency and myocardial perfusion, highlighting the importance of meticulous surgical technique and intraoperative assessment.

Distal anastomotic stenosis (shown by the red arrow) observed on postoperative angiography. This finding underscores the potential for technical issues at the distal anastomosis site, which may require percutaneous intervention or revision surgery.

Image illustrating an anastomosis performed before a significant vessel stenosis (shown by the red arrow). Suboptimal graft positioning or incomplete revascularization can contribute to ongoing myocardial ischemia and increase the likelihood of uCAG.

### 5.2. Patient-Related Factors Contributing to Ischemia

Besides surgical factors, incomplete revascularization and comorbidities such as diabetes, hypertension, and renal dysfunction exacerbate the risk of PMI [12]. Preußer et al. highlighted that these patient-specific risk profiles significantly correlate with uCAG findings post-CABG.

These conditions can impair endothelial function, promote inflammation, and increase the risk of thrombosis, all contributing to graft failure and myocardial ischemia. Careful management of these pre-existing conditions is essential for reducing the risk of PMI [14].

Fleißner et al. identified younger age, female gender, and combined arterial and venous revascularization as key risk factors for uCAG. Over half of these patients required repeat intervention, and in-hospital mortality remained high [11]. Also according to Preußer et al., smaller body size, female gender, and younger age have been associated with higher graft failure rates and the need for CAG, potentially linked to anatomical and vascular differences [13].

While the exact mechanisms underlying these associations are not fully understood, they may relate to differences in coronary artery anatomy, graft size, or hormonal factors. Further research is needed to clarify these relationships and develop targeted prevention strategies [4].

Incomplete revascularization occurs when not all significantly diseased coronary arteries are bypassed during the CABG procedure. This can leave areas of the myocardium vulnerable to ischemia, particularly if the native vessels progress or the bypass grafts fail [12].

### 5.3. Role of Inflammation and Thrombosis

Inflammation and thrombosis play a crucial role in the pathogenesis of postoperative myocardial ischemia. The inflammatory response triggered by CABG can promote thrombosis and graft occlusion, while platelet activation and the activation of the coagulation cascade contribute to early graft failure [21]. Surgical trauma, cardiopulmonary bypass, and ischemia–reperfusion injury can activate the inflammatory cascade, leading to the release of cytokines and other inflammatory mediators. These mediators can promote endothelial dysfunction, platelet activation, and thrombus formation, increasing the risk of graft failure. Antiplatelet and antithrombotic therapies are vital for preventing these complications. Acetylsalicylic acid (ASA), P2Y12 inhibitors, and other antiplatelet agents can reduce platelet activation and prevent thrombus formation. Anticoagulants, such as heparin or direct thrombin inhibitors, can inhibit the coagulation cascade and further reduce the risk of graft thrombosis [17].

## 6. Management Strategies Following Unplanned Coronary Angiography

The management of perioperative myocardial infarction detected by uCAG depends on the severity and underlying cause. Conservative therapy may suffice in minor cases or in the presence of reversible ischemia. PCI is now the preferred approach for most technical graft failures, as it offers effective revascularization with lower procedural risk than redo CABG [17]. Redo surgery remains reserved for cases where PCI is not feasible or has failed, such as in the setting of multiple occluded grafts or complex anatomy. Multidisciplinary decision making is essential to tailor therapy to each patient’s unique risk profile and anatomy.

### 6.1. Conservative Management

Conservative treatment is intended for minor graft issues or reversible ischemia. In such instances, medical management may effectively relieve symptoms and avert further myocardial damage. According to Heuts et al., around 43% of patients who underwent uCAG were treated medically without the need for intervention, often because of non-critical graft conditions like spasm or mild stenosis [7]. The management approach commonly involves beta-blockers, nitrates, and calcium channel blockers to optimize the balance between myocardial oxygen supply and demand [12]. Close monitoring remains vital to prevent further myocardial injury.

### 6.2. Percutaneous Coronary Intervention

PCI is the preferred method for addressing most graft failures detected by uCAG, facilitating direct revascularization with an acceptable level of procedural risk [13]. Research by Norman et al. indicated that 51% of patients who underwent uCAG received PCI treatment [2]. Nevertheless, performing PCI on graft lesions frequently necessitates longer fluoroscopy periods and the use of mechanical circulatory support, like intra-aortic balloon pump assistance, indicating the complexity of the procedure [19]. Thielmann et al. highlighted that PCI delivers better outcomes than redo surgery when addressing early graft failure [17]. According to Gaudino et al., uCAG following CABG can effectively identify graft failure in over 40% of patients, with PCI resolving ischemia in most instances; however, redo CABG presents a significantly elevated mortality risk [16]. Additionally, Norman et al. pointed out that 12% of graft failures can be detected within one week post-surgery if strict criteria for postoperative angiography are utilized [2].

### 6.3. Repeat Coronary Artery Bypass Grafting

Redo CABG is typically generally reserved for cases of anatomical failures where PCI is not feasible or has failed, such as with multiple graft occlusions, complex anatomy or severely compromised grafts [7]. Re-CABG is associated with a higher postoperative mortality rate in comparison to PCI or conservative management, primarily attributable to surgical complexity and patient vulnerability [12]. Davierwala et al. demonstrated that redo surgery frequently had prior unsuccessful PCI attempts, complicating management and outcomes [12]. Dell’Aquila et al. highlighted that, although PCI is commonly preferred for addressing graft failures, surgical reoperation remains a viable and secure option in selected cases, particularly when faced with anatomical complications such as graft kinking [24]. In order to equate procedural risks and advantages, multidisciplinary decision making that includes cardiologists, cardiac surgeons, and anesthesiologists is imperative.

## 7. Outcomes Associated with Unplanned Coronary Angiography

PMI after CABG is associated with a significantly poorer survival rate. One study reported that survival rates at 7 years were 62.5% in the PMI group and 81.1% in the non-PMI group [13]. Even when in-hospital deaths were excluded, survival rate differences remained significant, after correcting for the baseline differences between the PMI and non-PMI groups [13]. Davierwala et al., reporting a larger number of patients, observed a similar survival between discharged patients with PMI undergoing uCAG and those who showed no evidence of PMI [12,13]. This similar survival can be explained by the use of uCAG and consequent management for attenuating ischemia.

Angiography is a crucial diagnostic tool when PMI is suspected after CABG, helping to identify the underlying cause of ischemia, such as graft failure or native vessel occlusion [7,21]. The pooled early mortality rate after postoperative angiography, with or without repeat revascularization, has been reported as 8.9% [6]. This indicates that while CAG is essential for diagnosis and potential intervention, it is also associated with a notable risk of mortality in the early postoperative period.

In their meta-analysis, Biancari et al. ([6]) examined patients undergoing early angiography for suspected PMI after CABG [6]. They found that 31.7% of these patients had no angiographically detectable cause of ischemia, while 62.1% had acute graft failure. Notably, 46.3% of patients required repeat revascularization, most frequently with Redo CABG (54.2% vs. 45.8% for PCI). The same meta-analysis also demonstrated a protective influence of postoperative coronary angiography: in-hospital mortality in patients who suffered PMI and underwent reoperation without control angiography was 43.6%. Among these patients, acute graft failure was detected in 79.8% of cases. These findings highlight both the diagnostic and prognostic value of early postoperative angiography and underscore the importance of prompt and accurate assessment in this setting [6]. Introduction of early postoperative angiographic evaluation significantly reduced the rate of emergency reoperations (from 0.86 to 0.34%) and in-hospital mortality (from 46 to 22%), demonstrating the value of early and targeted diagnostics in patients with suspected ischemia after CABG [9]. In the cohort studied by Laflamme et al., early postoperative graft failure was diagnosed in 0.6% of CABG patients, and prompt re-intervention (PCI or redo CABG) was associated with a trend towards reduced myocardial injury compared with conservative management [10].

Patients undergoing uCAG due to PMI are more likely to require prolonged ventilation and reintubation [2]. These interventions are indicative of significant hemodynamic instability and respiratory compromise, highlighting the critical condition of patients requiring uCAG. The need for these supportive measures can prolong hospital stays and increase the risk of complications. Postoperative cardiac arrest and renal failure are also more frequent in this cohort [2]. Cardiac arrest reflects the severity of myocardial ischemia and the potential for life-threatening arrhythmias. Renal failure may result from hypoperfusion, nephrotoxic medications, or contrast-induced nephropathy, further complicating the patient’s recovery [2].

Several studies have shown that prompt diagnosis and treatment of bypass graft failure (either PCI or redo CABG) improve outcomes and can result in similar midterm survival for a non-ischemic patient [2,8,12]. Despite these improvements in midterm prognosis, the underlying cardiac disease and susceptibility to recurrent ischemia indicate that the long-term risk of cardiovascular events and mortality remains elevated in this population [7]. Heuts et al. prove that patients requiring uCAG for PMI are at higher risk of decreased long-term survival compared to those without postoperative ischemia [7]. These findings underscore the importance of early, aggressive management and long-term follow-up for this high-risk group.

Unplanned catheterization is associated with a significantly higher cost of care. The increased cost is related to higher complication rates and prolonged hospital stays. Patients undergoing uCAG due to PMI are more likely to require intensive care, mechanical ventilation, and other resource-intensive interventions [2]. These factors contribute to a substantial increase in the overall cost of their care.

Strategies to reduce the incidence of need for uCAG may lead to substantial cost savings [2]. By preventing postoperative myocardial ischemia and the need for uCAG, healthcare providers can reduce the burden on hospital resources and improve the cost-effectiveness of CABG procedures. This includes optimizing surgical techniques, implementing standardized monitoring protocols, and developing risk prediction models to identify patients at high risk for PMI [25].

In summary, uCAG and PMI are associated with increased short-term morbidity and mortality. However, expeditious identification and management of graft-related complications—particularly with PCI—can restore perfusion and improve midterm survival to levels approaching those of patients without ischemic complications. Long-term outcomes remain less favorable in this high-risk group, emphasizing the need for prevention, early detection, and individualized management throughout the perioperative period.

## 8. Strategies for Preventing Postoperative Myocardial Ischemia and Unplanned Coronary Angiography

Preventing postoperative myocardial ischemia requires a multifaceted strategy. Optimizing surgical technique—including careful graft handling, precise anastomosis, and intraoperative assessment using transit-time flowmetry (TTFM)—is essential for early detection and correction of technical issues. Pharmacologic prevention with antiplatelet agents, statins, and vasodilators helps maintain graft patency and reduce the risk of early graft spasm or thrombosis. Standardized protocols for postoperative monitoring, early detection of ischemic changes, and prompt intervention remain central to improving outcomes [18,26,27].

### 8.1. Optimizing Surgical Technique

Meticulous surgical technique is critical for ensuring graft patency and preventing premature graft failure [21]. This involves the diligent handling of grafts, precise construction of anastomoses, and meticulous attention to detail in all aspects of the surgical procedure. By minimizing technical errors, surgeons can mitigate the risk of graft failure and postoperative ischemia. The construction of anastomosis should be executed with precision to facilitate a smooth, patent connection between the graft and the coronary artery. Grafts should be handled with care to prevent damage or kinking, which could jeopardize blood flow. Intraoperative transit-time flow measurement can be employed to evaluate graft flow during surgery and to identify any technical inaccuracies that may be undermining graft patency. Rectifying these inaccuracies intraoperatively can enhance long-term outcomes and diminish the risk of postoperative ischemia.

Intraoperative graft flow assessment using transit-time flowmetry (TTFM) has become an increasingly valuable tool for quality control during CABG procedures. TTFM provides real-time, quantitative evaluation of graft blood flow, enabling the surgical team to immediately detect technical issues such as kinking, anastomotic stenosis, or competitive flow. Early identification and correction of these problems can significantly reduce the incidence of postoperative myocardial ischemia and may decrease the necessity for uCAG. Recent studies and expert consensus statements support the routine use of TTFM as a means to improve graft patency and overall surgical outcome [18,26,27].

### 8.2. Pharmacological Therapy

In addition to technical measures, perioperative pharmacologic therapy plays an important role in the prevention of myocardial ischemia after CABG.

Antiplatelet therapy utilizing ASA and/or P2Y12 inhibitors is essential for the prevention of graft thrombosis [17]. ASA is generally commenced preoperatively, while P2Y12 inhibitors may be introduced postoperatively in selected patients.

Agents such as diltiazem, a calcium channel blocker, and nitroglycerin, a vasodilator, are commonly used to prevent and treat coronary or graft spasm, which can contribute to early graft failure [18,21]. Nitrates facilitate the relaxation of smooth muscle in the coronary arteries and bypass grafts, thereby enhancing blood flow to the myocardium. Nitrates can be administered intravenously or topically to manage graft spasm and avert postoperative ischemia. Early administration of these agents in high-risk patients may help maintain graft patency, optimize myocardial perfusion, and potentially reduce the incidence of postoperative ischemic events requiring uCAG. These strategies should be tailored to individual patient risk profiles, and their benefits have been supported by several observational and randomized studies.

Statins have been demonstrated to enhance endothelial function and diminish the risk of graft failure. Research indicates that statins can improve endothelial function, reduce inflammation, and stabilize atherosclerotic plaques. These effects collectively contribute to decreasing the risk of graft failure and postoperative ischemia [17].

### 8.3. Monitoring and Early Detection Protocols

The implementation of standardized protocols for the monitoring of cardiac biomarkers and electrocardiogram (ECG) changes can significantly enhance the early detection of post-myocardial infarction (PMI) [22]. Consistent monitoring of cardiac biomarkers, including troponin and CK-MB, is instrumental in identifying myocardial injury at an early stage within the postoperative period. Additionally, ECG alterations, such as ST-segment elevation or depression, may serve as indicators of myocardial ischemia.

The regular assessment of hemodynamic parameters and clinical status is paramount. Monitoring hemodynamic metrics, including blood pressure and heart rate, aids in identifying patients exhibiting reduced cardiac output or hemodynamic instability. Furthermore, systematic evaluation of clinical status, encompassing symptoms such as chest pain, shortness of breath, and other relevant indications, can facilitate the early detection of PMI throughout its progression.

Establishing explicit criteria for initiating further investigations, which may include uCAG, can enhance patient outcomes.

## 9. Challenges and Controversies Surrounding Unplanned Coronary Angiography

One of the primary challenges in the field is the lack of standardized criteria for defining PMI and determining the need for uCAG [7]. This heterogeneity leads to variations in practice, making it difficult to compare outcomes across different studies.

Unplanned CAG is an invasive procedure with inherent risks, including bleeding, infection, and contrast-induced nephropathy [15]. Balancing these risks with the potential benefits of identifying and treating correctable causes of ischemia is a critical consideration.

The optimal timing of uCAG remains a topic of debate. Some studies suggest that prompt management of PMI is associated with better outcomes [13], while others indicate that delaying angiography beyond a certain threshold may worsen outcomes. Davierwala et al. reported that postoperative coronary angiography performed more than 30 h after CABG resulted in a significantly worse outcome [12].

The decision to pursue conservative management versus interventional strategies (PCI or redo surgery) based on unplanned CAG findings is another area of controversy. While revascularization may improve outcomes in some patients, it also carries additional risks and costs [12].

## 10. Future Directions and Research Needs

Emerging non-invasive techniques such as cardiac magnetic resonance imaging (CMRI) and computed tomography angiography (CTA) are promising tools for early detection of graft dysfunction and myocardial ischemia, potentially reducing reliance on invasive CAG in selected patients [19]. However, further validation of these methods in the immediate post-CABG setting is essential, particularly to determine their diagnostic accuracy, cost-effectiveness, and impact on patient outcomes.

Another area of future research is the development and refinement of risk prediction models for perioperative myocardial infarction and unplanned CAG. Improved models that integrate preoperative, intraoperative, and postoperative variables—including patient demographics, operative details (such as cross-clamp time and cardioplegia type), and dynamic biomarker trends—may enable better identification of high-risk patients. Robust external validation across diverse populations is needed to ensure clinical utility and generalizability of these tools [7].

There is also a pressing need for high-quality randomized controlled trials (RCTs) to compare different management strategies for PMI and uCAG [18,23]. Such trials should evaluate the relative benefits and risks of PCI, redo CABG, and conservative medical therapy, as well as the optimal timing of intervention (early versus delayed strategies). These studies would provide much-needed evidence to inform future guidelines and optimize both short-term and long-term patient outcomes [7,19]

Additionally, advances in intraoperative monitoring—such as routine use of transit-time flowmetry and emerging real-time imaging techniques—warrant further investigation for their potential to prevent technical graft failure. Large, prospective multicenter registries will be valuable to track outcomes and identify practice patterns across institutions.

Finally, the integration of artificial intelligence and machine learning into perioperative data analysis may enhance risk stratification and support personalized management approaches in this complex patient population.

Together, these research directions have the potential to substantially improve the detection, management, and prognosis of perioperative myocardial infarction and unplanned postoperative coronary angiography following CABG.

## 11. Conclusions

Unplanned CAG following isolated CABG remains a clinically relevant challenge with significant implications for early morbidity and mortality. The timely identification and management of PMI are essential, as early intervention can improve midterm outcomes. Our review highlights the variability in diagnostic criteria and institutional protocols, emphasizing the need for greater standardization in both the definition and management of PMI after CABG.

Looking ahead, optimizing intraoperative techniques—including the routine use of graft flow measurement—combined with vigilant postoperative monitoring and tailored pharmacologic prevention may reduce the incidence of postoperative ischemia and the need for uCAG. Furthermore, establishing standardized definitions and clinical pathways for the diagnosis and management of PMI will be essential for enhancing long-term outcomes and mitigating healthcare costs. Future research should focus on refining risk stratification models, validating non-invasive diagnostic tools, and comparing management strategies in prospective studies to further improve patient outcomes and healthcare efficiency in this complex setting.

When appropriately indicated and executed without delay, unplanned CAG presents an invaluable opportunity to salvage myocardium, avert irreversible damage, and ultimately improve the prognosis of patients undergoing coronary surgery procedures.

## Figures and Tables

**Table 1:** Incidence of unplanned postoperative coronary angiography after coronary artery bypass grafting in selected studies.

Study/Source	Study Design	Incidence (%)	Sample Size	Notes/Population
Norman et al. (2024) [2]	Single-center, retrospective	0.39	7011	Isolated CABG, strict criteria, early CAG
Heuts et al. (2024) [7]	Multicenter, observational	4.1	2591	Excluded planned hybrid procedures
Davierwala et al. (2013) [12]	Single-center, prospective	5.3	1026	Non-emergency isolated CABG
Preußer et al. (2018) [13]	Single-center, retrospective	4.2	1206	Early CAG for suspected PMI
Biancari et al. (2018) [6]	Meta-analysis	4.4 (pooled)	6774	Multiple studies, diverse populations
Alqahtani et al. (2019) [14]	Nationwide registry (US)	2.5	72,241	In-hospital PCI post-CABG
Rupprecht et al. (2019) [15]	Single-center, observational	2.3	3136	Early CAG post-CABG
Fleißner et al. (2017) [11]	Single-center, retrospective	1.8	6025	Urgent CAG after CABG/valve
Gaudino et al. (2015) [16]	Single-center, retrospective	2.6	2202	Post-cardiac surgery, including CABG
Hultgren et al. (2016) [8]	Single-center, retrospective	2.0	4446	Acute CAG within 48 h post-CABG
Karhunen et al. (2010) [9]	Single-center, prospective	0.7	5251	Early angiography for persistent ischemia
Laflamme et al. (2012) [10]	Single-center, retrospective	0.7	5598	Early CAG for ischemia; 32/5598 had early graft failure
Thielmann et al. (2006) [17]	Single-center, prospective	2.2	5427	Repeat CAG for suspected perioperative MI within 24 h

## Abbreviations

ASA	Acetylsalicylic acid
CABG	Coronary artery bypass grafting
uCAG	Unplanned coronary angiography
PCI	Percutaneous coronary intervention
PMI	Postoperative myocardial ischemia
CMRI	Cardiac magnetic resonance imaging
CTA	Computed tomography angiography
CK-MB	Creatine kinase-MB
hs-cTnI	High-sensitivity cardiac troponin I
RCT	Randomized controlled trial
